# Classic Kaposi Sarcoma in an Immunocompetent Male From Haiti: An Unusual Epidemiological Presentation

**DOI:** 10.7759/cureus.64518

**Published:** 2024-07-14

**Authors:** Emanuel Vanegas, Aye M Thida, Edward Heilman, Mohan Preet

**Affiliations:** 1 Department of Internal Medicine, Woodhull Medical Center, New York, USA; 2 Department of Hematology and Oncology, State University of New York (SUNY) Downstate Health Sciences University, New York, USA; 3 Department of Dermatology and Pathology, State University of New York (SUNY) Downstate Health Sciences University, New York, USA

**Keywords:** kaposi sarcoma management, kaposi sarcoma treatment, kaposi tumor, kaposi sarcoma in an immunocompetent patient, kaposi sarcoma hiv negative

## Abstract

Kaposi sarcoma (KS) is an angioproliferative disorder caused by human herpesvirus-8 (HHV-8) infection. KS manifests as vascular and mucosal nodules and is classified into four subtypes based on epidemiology, clinical presentation, histopathology, and HHV-8/human immunodeficiency virus serology. Here, we present a unique case of classic KS in an 84-year-old immunocompetent Haitian male patient, highlighting the rarity of this variant in this population. Additionally, our article delves into the broader context by reviewing a few documented cases of classic KS in the Caribbean region.

## Introduction

Kaposi sarcoma (KS) is an angioproliferative disorder resulting from human herpesvirus-8 (HHV-8) infection, which commonly manifests as vascular and mucosal nodules. Based on epidemiology, clinical presentation, histopathology, and HHV-8/human immunodeficiency virus (HIV) serology status, KS can be classified into four main subtypes: classic KS, endemic or African KS, epidemic or acquired immune deficiency syndrome (AIDS)-associated KS, and iatrogenic KS.

Infection with HHV-8 is required for KS, as it is directly involved in the pathogenesis of all subtypes of KS [1]. Humans serve as natural hosts for HHV-8, which is spread through saliva and sexual contact and can remain latent in CD19+ B cells [1,2]. The KS workup includes a biopsy for a definitive diagnosis, laboratory tests to determine serology, immune status, or other hematological abnormalities, and imaging if a distant spread is suspected. 

The first description of KS was made by Moritz Kaposi in 1872 [3]. It was first described as a tumor of the lower extremity in elderly people. In the modern era, this presentation is referred to as classic KS, commonly seen in men of Eastern European, Mediterranean, and Middle Eastern descent [4,5]. This report describes the case of an 84-year-old immunocompetent Haitian male patient with classic KS. To our knowledge, no classic variant cases have been reported in this population. A few cases of classic KS have been reported in other Caribbean regions, which are briefly reviewed in this article. 

## Case presentation

An 85-year-old male patient presented with a painful rash on his right lower extremity, which was later accompanied by a serosanguineous discharge that eventually prevented him from walking. He has also had multiple episodes of cellulitis over the last decade, the most recent of which was associated with sepsis. His past medical history includes hypertension, diabetes, and asthma. He has never smoked, drinks alcohol socially, and does not use drugs. Originally from Haiti, the patient migrated to the United States 20 years ago and worked as a wall painter for over 25 years. 

**Figure 1:**
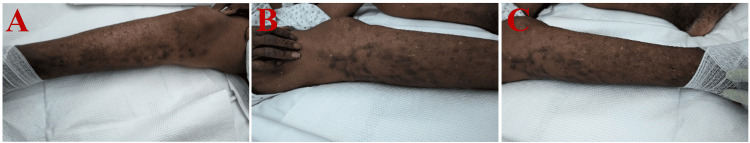
Clinical pictures A)-C) Violaceous, dark-brown patches and macules on the inner aspect of the right lower extremity; hyperpigmentation on the inner aspect of the left ankle

On physical examination, there were violaceous, dark-brown patches and macules, most prominent on the distal right lower extremity, which extended proximally up to the knee (Figures 1A-1C). The left lower extremity exhibited a few macules, and both ankles were hyperpigmented. Apart from that, the examination was unremarkable. 

**Figure 2:**
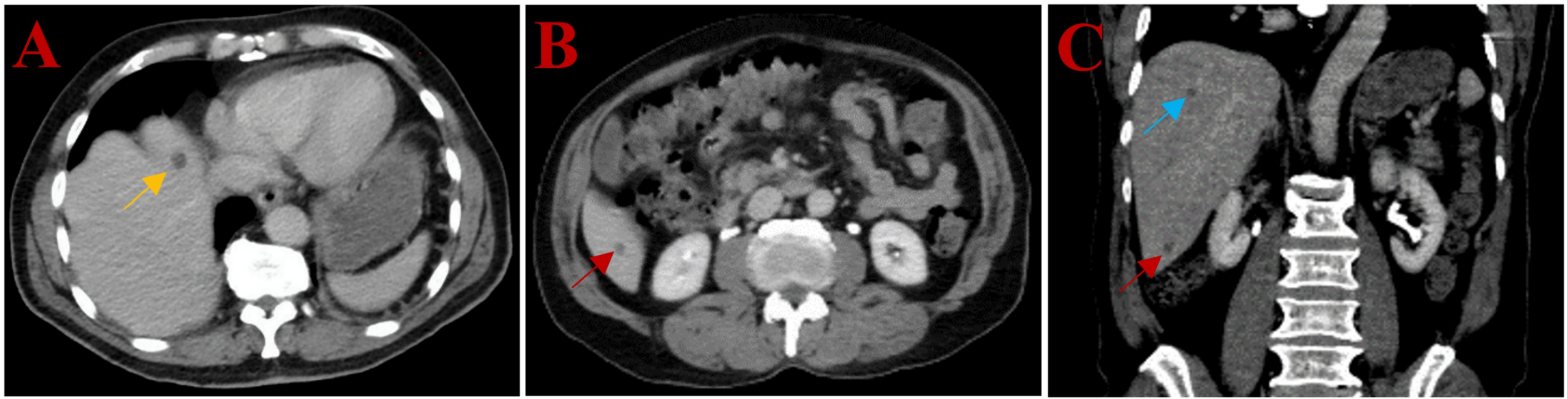
Computed tomography imaging A) CT scan of the abdomen without contrast, axial view demonstrating left hepatic lobe hypoattenuation measuring 1.1 cm in largest axis diameter (yellow arrow); B) CT scan of the abdomen without contrast, axial view demonstrating right hepatic lobe hypoattenuation near the inferior hepatic border (red arrow); C) CT scan of the abdomen without contrast, coronal view demonstrating hypoattenuations in the right lobe (blue and red arrows) CT: Computed tomography

Laboratory results were notable for mild anemia, with a hemoglobin level of 10.4 g/dL. The HIV test was negative, and the T-cell subset and total immunoglobulin levels were normal. A right lower extremity computed tomography (CT) scan with intravenous contrast showed increased subcutaneous edema and skin thickening at the knee level. It also revealed a soft tissue density within the inner thigh subcutaneous fat and along the plantar surface of the foot. There was no fluid collection. Furthermore, several prominent lymph nodes, with the largest measuring 1.1 cm, were identified in the right inguinal region. A bilateral duplex ultrasound did not detect any vascular abnormalities. A CT scan of the abdomen and pelvis revealed multiple hypoattenuating hepatic lesions, the largest measuring up to 1.1 cm in the left lobe (Figures 2A-2C). The CT scan of the chest was unremarkable.

**Figure 3:**
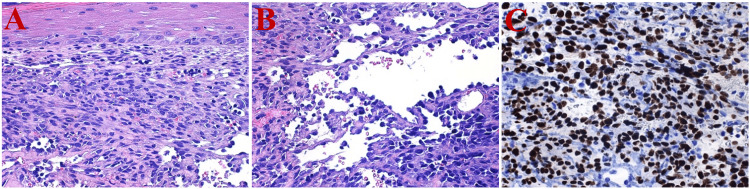
Punch biopsy pathology A) Hematoxylin and eosin staining at an original magnification of 400x shows a densely packed proliferation of hyperchromatic spindle-shaped cells forming slit-like clefts filled with erythrocytes; B) Hematoxylin and eosin staining at an original magnification of 400x reveals a focus with a lymphangioma-like pattern; C) Positive HHV-8 immunoenzyme stain (400x) HHV-8: Human herpesvirus-8

A punch biopsy was performed, which revealed a patch-plaque stage KS (Figures 3A-3B). Immunostaining for HHV-8 was positive (Figure 3C). Periodic acid-Schiff stain, Grocott methenamine silver stain, and acid-fast bacilli stain were negative. 

## Discussion

Incidence of classic KS is higher in males than in females, with a 17:1 ratio [6]. A majority of cases have been reported among individuals of Eastern European, Mediterranean, and Middle Eastern descent [1,5]. Contrary to AIDS-associated KS, which typically presents in young to middle-aged adults, classic KS usually peaks in the sixth to seventh decade of life [5,7]. We present the case of an 84-year-old Haitian man. At the present time, there is no epidemiologic evidence that the Caribbean region is associated with classic KS. In fact, few cases have been reported in this region, which are summarized in Table 1 [8-10].

**Table 1:** Demographic and clinical characteristics of patients with classic Kaposi sarcoma reported in Caribbean countries M: Male; F: Female; HT: Heterosexual; MSM: Men who have sex with men; NR: Not reported; IHC: Immunohistochemistry; HHV-8: Human herpesvirus-8; HIV: Human immunodeficiency virus; PCR: Polymerase chain reaction

Country	Patients	Reference	Patient's demographics (age/gender/sexual orientation)	Cutaneous involvement	Mucosal/visceral involvement	HHV-8/HIV status (method)	CD4 cell count (cells/mm^3^)
Dominican Republic	1	[8]	55/M/NR	Bilateral lower extremities; Right: clustered dark nodular lesions on medial thigh; purple plaques below the knee. Left: purple plaques below the knee; edema	Mucosal involvement: Yes; violaceous lesion of the soft palate. Visceral involvement: No	HHV-8: Confirmed (IHC); HIV: Negative (serology)	350
Cuba	4	[9]	61/M/HT	Unilateral right lower extremity; maculopapular purple tumor on plantar region; edema	Mucosal involvement: No; Visceral involvement: No	HHV-8: Confirmed (Quantitative PCR); HIV: Negative (NR)	1058
			39/M/MSM	Unilateral right lower extremity; 5 mm lesion on knee	Mucosal involvement: No; Visceral involvement: No	HHV-8: Confirmed (Quantitative PCR); HIV: Negative (NR)	783
			58/M/HT	Unilateral left lower extremity; purple lesion on heel	Mucosal involvement: No; Visceral involvement: No	HHV-8: Confirmed (Quantitative PCR); HIV: Negative (NR)	NR
			64/F/HT	Bilateral upper extremities; Right: maculopapular lesions on forearm. Left: two small lesions on arm	Mucosal involvement: No; Visceral involvement: No	HHV-8: Confirmed (Quantitative PCR); HIV: Negative (NR)	NR
French Guiana	1	[10]	NR	NR	NR	HHV-8: NR; HIV: Negative (NR)	NR

KS was first documented in Haiti in 1972, following the autopsy of a 52-year-old male whose course of illness is unknown [11,12]. No other cases were identified until 1979, when an apparent outbreak of acquired T-cell immunodeficiency syndrome presenting with KS or other opportunistic infections was identified, with Port-au-Prince being the most affected location. Three publications documented this outbreak from 1979 to 1982 and described patients in their mid-30s, with lymphatic and visceral involvement and a poor prognosis regardless of their sexual orientation or socioeconomic status [11-13]. Since the first HIV-antibody screening test in blood was not available until 1985, it was not possible to confirm whether these patients had HIV infection. While speculative, it is likely that these were epidemic cases of KS. Furthermore, given the young onset of these cases and the fact that nearly 95% of Haitians are of African descent, with 45% from sub-Saharan Africa, endemic KS variants may also have been present. 

The presentation of KS in HIV-negative Haitian patients has been described as more atypical compared to HIV-positive Haitian patients. Poniecka et al. described a 45-year-old HIV-negative female patient with a slowly enlarging left thyroid mass, without other symptoms [14]. Kotzias et al. described a 63-year-old male presented with exophytic ulcerations covering over 60% of his left lower extremity, without any systemic involvement [15]. Pathology was compatible with lymphangioma-like classic KS, a cutaneous variant that can occur in any subtype of KS.

In contrast, classic KS is characterized by an indolent course and is distinguished by violaceous or reddish macules, patches, or plaques, with or without nodules. The nodules may become ulcerated and bleed. Most commonly, these cutaneous lesions occur in the lower extremities, followed by the upper extremities, head, and trunk. Although these lesions are generally asymptomatic, patients may experience mobility and quality of life impairments if associated with lymphedema. Upon presentation, our patient appeared with bilateral lower extremity macules, patches, plaques, and nodules, as well as weight-bearing pain that had resulted in difficulty walking. Whether these nodules had ulcerated in the past is unknown. A review of the patient's chart, however, revealed that he had had recurrent episodes of "purulent" discharge on the right lower extremity for over 14 years that had not improved with antibiotic treatment. Many of these episodes resolved spontaneously, a phenomenon seen in classic KS that has been reported elsewhere [16]. KS has also been identified as a risk factor for recurrent cellulitis, which is most often associated with moderate to severe lymphedema [17]. Although our patient presented with indurated and hyperpigmented skin, which was indicative of chronic disease, there were few signs of swelling upon examination. 

Distant metastases are uncommon in classic KS. Dissemination and rapid spread are often associated with immunosuppression; the latter was not present in this case. The most common sites of involvement are the gastrointestinal tract and lymph nodes. When metastases are suspected in patients with a long life expectancy, appropriate staging workups, such as colonoscopy, endoscopy, and CT scans of the chest, abdomen, and pelvis with intravenous contrast, should be considered. Our patient underwent CT scans of the chest, abdomen, and pelvis with intravenous contrast and was found to have bilateral inguinal lymph node involvement and possible metastatic liver disease. Biopsies may be beneficial in patients with KS spread to distant sites, particularly when they are asymptomatic, as this can assist in guiding their treatment. In particular, patients with focal skin disease without involvement of viscera or lymph nodes may benefit from topical or local chemotherapy. Alternatively, systemic chemotherapy should be considered whenever a patient has (I) symptomatic disease with extensive skin spread, (II) visceral involvement regardless of symptomatology or surface involvement, or (III) disease that is rapidly progressing [18]. Considering that our patient met the first criterion for systemic chemotherapy, as well as probably the remaining two, a liver or lymph node biopsy was not performed, as it would not have changed his treatment plan. 

Several strategies for systemic chemotherapy have been studied in classic KS, either in combination or as a single agent. Due to the rarity of classic KS, a limited number of prospective clinical trials have been conducted in this population. Consequently, most of the current treatment recommendations are based on studies carried out on patients with HIV-associated KS. Early on, combinations of bleomycin and vincristine or doxorubicin, bleomycin, and vincristine were widely used due to their reported response rates of 57% to 88% [19-21]. Unfortunately, combination regimens usually result in greater toxicity. Therefore, current guidelines recommend pegylated liposomal doxorubicin (PLD) as a single agent in the absence of contraindications, as it is well tolerated and achieves comparable response rates to combination therapies, if not better. For example, in randomized clinical trials involving patients with HIV-associated KS, liposomal doxorubicin alone achieved response rates between 46% and 58%, compared to 25% with combination regimens [22,23]. Further, an even higher response rate has been reported for patients with classic KS, although these data are drawn from retrospective studies. For instance, a multicenter analysis of patients without prior systemic chemotherapy reported an overall response rate with PLD of 71% and disease control for more than two years following treatment initiation [24]. Two retrospective studies examined PLD as second-line therapy with similar results [25,26]. 

A number of second-line drugs are also available as single-agent regimens, including paclitaxel, etoposide, vinblastine, vinorelbine, and gemcitabine. Among these, paclitaxel has been the most studied, given its high response rate and tolerable side effects. In two case series, paclitaxel was found to be effective in treating refractory or life-threatening classic KS, with an overall response rate exceeding 95% [27,28]. A retrospective study of 58 patients with stage IIB to IV/anaplastic HIV-negative KS showed that a weekly dose of 100 mg of paclitaxel resulted in a partial or complete response rate of 94.6% by the end of the first cycle [29]. In a case series involving 17 patients with advanced, aggressive classic KS, the same dose was found to be effective and well tolerated [30]. The patient presented in this case was a suitable candidate for single-agent therapy with PLD; however, he declined to undergo therapy. 

## Conclusions

Classic KS is relatively uncommon and has been found more frequently in individuals of Eastern European, Mediterranean, and Middle Eastern descent. It can present diagnostic challenges, especially for individuals who do not fit the typical demographic profile. This case report demonstrates the importance of recognizing that classic KS can still be present in immunocompetent patients of all ethnic groups. Early diagnosis of classic KS is crucial and can be facilitated by maintaining a high suspicion index and utilizing imaging and biopsy techniques. Once diagnosed, prompt intervention with localized or systemic therapy can be initiated, which may offer potential benefits for treatment outcomes and the overall well-being of the patients.
